# Therapeutic Properties of Mesenchymal Stromal/Stem Cells: The Need of Cell Priming for Cell-Free Therapies in Regenerative Medicine

**DOI:** 10.3390/ijms22020763

**Published:** 2021-01-14

**Authors:** Vitale Miceli, Matteo Bulati, Gioacchin Iannolo, Giovanni Zito, Alessia Gallo, Pier Giulio Conaldi

**Affiliations:** Research Department, IRCCS ISMETT (Istituto Mediterraneo per i Trapianti e Terapie ad alta Specializzazione), 90127 Palermo, Italy; mbulati@ismett.edu (M.B.); giannolo@ismett.edu (G.I.); gzito@ismett.edu (G.Z.); agallo@ismett.edu (A.G.); pgconaldi@ismett.edu (P.G.C.)

**Keywords:** Mesenchymal stromal/stem cells, priming, paracrine mechanism, cell-free therapies, regenerative medicine

## Abstract

Mesenchymal stromal/stem cells (MSCs) are multipotent adult stem cells that support homeostasis during tissue regeneration. In the last decade, cell therapies based on the use of MSCs have emerged as a promising strategy in the field of regenerative medicine. Although these cells possess robust therapeutic properties that can be applied in the treatment of different diseases, variables in preclinical and clinical trials lead to inconsistent outcomes. MSC therapeutic effects result from the secretion of bioactive molecules affected by either local microenvironment or MSC culture conditions. Hence, MSC paracrine action is currently being explored in several clinical settings either using a conditioned medium (CM) or MSC-derived exosomes (EXOs), where these products modulate tissue responses in different types of injuries. In this scenario, MSC paracrine mechanisms provide a promising framework for enhancing MSC therapeutic benefits, where the composition of secretome can be modulated by priming of the MSCs. In this review, we examine the literature on the priming of MSCs as a tool to enhance their therapeutic properties applicable to the main processes involved in tissue regeneration, including the reduction of fibrosis, the immunomodulation, the stimulation of angiogenesis, and the stimulation of resident progenitor cells, thereby providing new insights for the therapeutic use of MSCs-derived products.

## 1. Introduction

**Figure 1:**
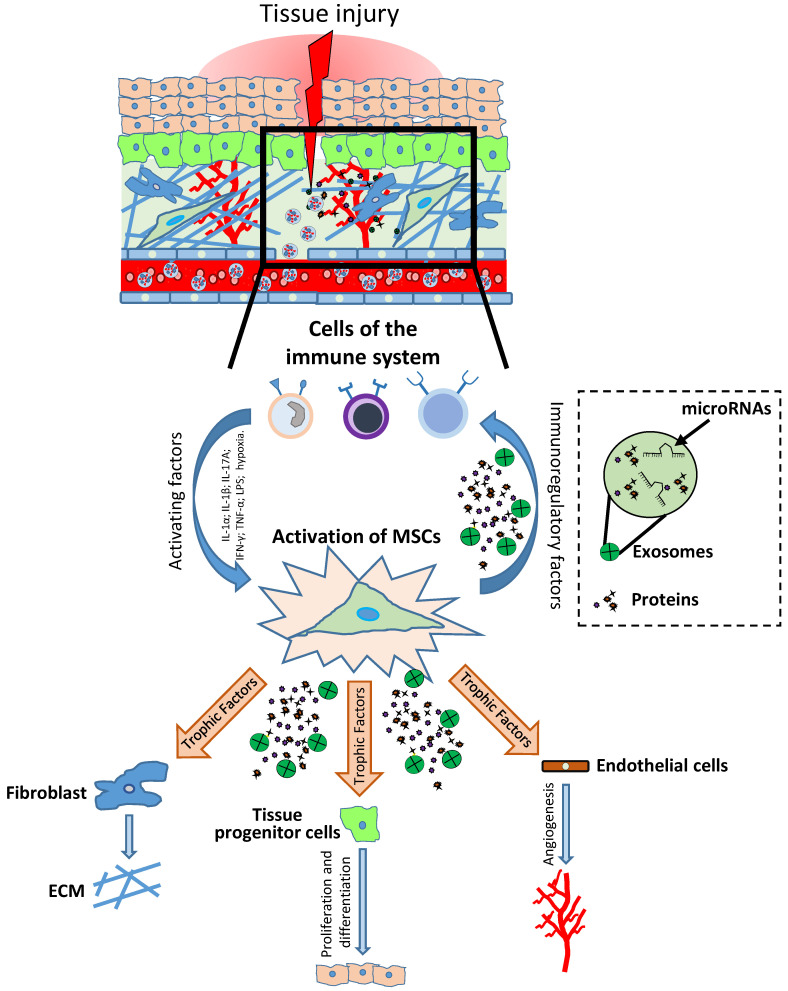
Role of mesenchymal stromal/stem cells (MSCs) in tissue injury and repair. After injury, the damaged tissue activates MSCs through different inflammatory signals (IL-1β, IFN-γ, TNF-α, LPS; hypoxia). MSC activation leads to coordination of the microenvironment by both the production of immunomodulatory factors (to modulate the progression of inflammation) and the production of growth factors which subsequently stimulate endothelial cells, fibroblasts cells and tissue progenitor cells. The physiological and orderly action of these factors allows tissue repair through angiogenesis, remodeling of the extracellular matrix (ECM) and functional tissue restoration through tissue progenitor cells differentiation.

Mesenchymal stromal/stem cells (MSCs) belong to the pool of adult stem cells that, in a specific microenvironment termed the “stem cells niche”, support tissue regeneration in both physiologic and pathologic conditions contributing to tissue homeostasis [1,2,3]. These cells can increase their own compound [4] and replace individual components of the microenvironment by differentiating or attracting supporting cells to a niche [1,2,3]. It has been shown that in tissues such as intestine and skin, MSCs support a high cellular turnover [5,6]. In contrast, in other tissues such as skeletal muscle, MSCs are considered adult stem cells that support regeneration after injury, even if they marginally contribute to myofiber renewal during physiologic turnover [7]. Indeed, MSC-like cells with adipogenic phenotype resident in the muscles are quiescent in intact tissue, but get activated in injured ones, providing a momentary source of key factors that induce proliferation of myogenic progenitor cells. Thus, these MSCs that normally have adipogenic potential, when injury-activated, can stimulate the differentiation of the myogenic progenitor’s cells supporting tissue repair [3,8]. A similar phenomenon has been shown in skin tissue, where MSC-like adipose precursor cells within the skin appear to be crucial for epithelial cell regulation [9]. Therefore, MSCs can be considered as key regulatory components in the regenerating stem cell niche (Figure 1).

These findings provide a robust rationale to investigate the role of MSCs as a therapeutic product to support tissue injury responses in different diseases. MSCs are multipotent cells with easy accessibility, few ethics-related issues, and higher adaptability to in vitro cultures for expansion. Unlike pluripotent stem cells, multipotent MSCs have a limited self-renewal capacity [10]. In light of this, in recent years the “stem” cell definition has been changed to “stromal” in order to give a more appropriate connotation to describe MSCs. Furthermore, these cells are immuno-privileged due to their low expression of *CD40*, *CD80*, *CD86* and major histocompatibility complex I (*MHC I*), as well as the lack of *MHC II* expression [11,12,13,14]. These features make these cells a very useful tool for cell therapy in the field of regenerative medicine.

MSCs are found in several tissues, including bone marrow (BM) [15], adipose tissue (AT) [16], umbilical cord (UC) [17], dental pulp [18] and placenta [19], where these cells are surrounded by different cell types such as immune cells, epithelial cells, endothelial cells and stromal cells, and can exhibit immunomodulatory [20,21], angiogenic [22,23] and anti-oxidative properties [24]. Over the past decade the therapeutic action of MSCs has been investigated in several clinical trials for the treatment of many disorders including cardiovascular, neurodegenerative, immune, lung, liver, kidney and orthopedics diseases (clinicaltrials.gov). In these cases, MSCs have been shown to have moderate or poor efficacy, and the results from different clinical trials are controversial [25,26,27,28,29], indicating an urgent need to optimize the therapeutic use of MSCs or to enhance MSC potency. This inconsistent evidence is potentially related both to intrinsic differences in the use of cell-based products and to the lack of standardized methods for MSC production that affects their potency. MSC effects depend both on tissue source [30,31] and on how they are produced and administered. Indeed, it has been shown that the composition of MSCs secretome can be modulated by preconditioning of MSCs with hypoxia and cytokines treatments, as well as the growing of MSCs under specific culture systems, including three-dimensional (3D) culture conditions [32,33,34,35]. In response to MSC “priming”, the production of factors is switched towards an anti-inflammatory and pro-trophic phenotype that results in a homeostatic regulation of tissue regeneration/repair [36,37]. Currently, it is often stated that the efficacy of MSCs therapies is probably not related to cell engraftment and replacement but is linked to the production of crucial paracrine factors, such as cytokines, growth factors, and exosomes (EXOs), that regulate the cell niche for their regeneration. Indeed, in response to specific stimuli, MSCs are activated and can secrete a plethora of regulating factors that affect tissue injury responses in a transitory and paracrine manner to orchestrate the repairing tissue processes [20,38,39,40,41,42,43,44]. In a different model of injury it has been shown that MSCs, mainly triggered by inflammation processes, induce tissue regeneration/repair by cell niche empowerment/regulation [45,46,47]. In these cases, in an inflammatory-injured tissue, MSC effects were mediated by paracrine mechanisms that lead to regulation of fibrosis, immunomodulation, stimulation of angiogenesis and stimulation of resident cells to coordinate both tissue regeneration and function recovery [37,48,49,50,51,52]. Therefore, due to the regenerative potential and trophic properties of specific MSC-derived products, such as the conditioned medium (CM) and EXOs, these products have emerged as possible therapeutic tools with numerous applications and are consequently being extensively evaluated for medical use [53,54,55]. In addition, the clinical application of MSC-derived products must be considered for their advantages as opposed both to the lack of safety in the long-term use of MSCs and the risks related to transmission of infection diseases, such as some viruses found in the transplanted allogenic cells.

In order to make the clinical application of MSC-derived products advanced in the field of regenerative medicine, the first point is to optimize the therapeutic strategies by the identification of the best way to prime MSCs and to improve their regenerative properties. This review focuses on promising cell priming methods that enhance paracrine therapeutic properties of MSCs in the main processes of tissue regeneration, such as angiogenesis, immunomodulation, fibrosis and stimulation of tissue resident cells.

## 2. Main MSC Priming Strategies to Enhance the Production of Key Factors that Stimulate Resident Cells for Tissue Regeneration/Repair

As mentioned above, MSC preconditioning has been considered an important tool to improve the effects of MSCs in regeneration and repair of injured tissues. The different priming strategies have been implemented according to the cell types that needed to be targeted in the injured tissues. Indeed, while the priming of MSCs with pro-inflammatory cytokine and 3D cultures has been mainly tested to modulate the inflammation and stimulate the angiogenesis in injured tissues, hypoxia has been applied as the major priming method of MSCs in order to enrich their CM with soluble factors involved in the stimulation of resident cells, such as parenchymal and tissue-specific stem cells. Thus, in the following paragraph we will focus our attention on the literature defining the role of preconditioned MSCs and the identified soluble factors which were associated with the stimulation of tissue-specific resident cells.

It has been shown that in hypoxia conditions MSCs up-regulated several factors that contributed to hepatocyte proliferation in vitro and liver regeneration in vivo in hepatectomized mice [56]. In this work, Lee and collaborators demonstrated that the MSC-derived CM was enriched in factors including interleukin 6 (IL-6), tumor necrosis factor alpha (TNF-α), hepatocyte growth factor (HGF) and vascular endothelial growth factor (VEGF), and this secretome was able to increase proliferating cell nuclear antigen (*PCNA*) and *Ki67* expression as well as markers of liver regeneration, such as signal transducers and activators of transcription 3 (pSTAT3), and down-regulation of suppressor of cytokine signaling 3 (SOCS3) [56]. Similarly, Yu and colleagues showed that the CM derived from hypoxia-induced MSCs promoted liver regeneration through VEGF signaling [57]. Another study from Leroux and co-workers showed that hypoxia preconditioning of MSCs improved the survival of engrafted MSCs in a mouse hindlimb ischemia model. Furthermore, the authors clearly showed that hypoxic MSCs, among others, stimulated the differentiation of resident myoblast via Wnt4 soluble factor release in a paracrine manner [58]. Recently, in an in vivo model of myocardial infarction, Hu and co-workers demonstrated that hypoxia preconditioned MSCs inhibited cardiac apoptosis and stimulated cardiomyocytes proliferation [59]. Furthermore, it has been shown that EXOs from hypoxic MSCs were enriched in miR-26a which, in turn, activated Wnt signaling to promote cardiomyocyte survival in a rat model of cardiac ischemia-reperfusion [60].

Hypoxia priming of MSCs has been shown to promote neurogenesis in a rat model of traumatic brain injury. In particular, Chang and colleagues demonstrated that the CM derived from hypoxia-treated MSCs was enriched in soluble factors including HGF and VEGF which, in turn, stimulated the proliferation of neuronal cells in rat peri-ischemic brain regions [61]. However, the author speculated that other undefined soluble factors in the hypoxic preconditioned MSC CM might be involved in the rescue of the neural phenotype. In a similar manner, it has recently been demonstrated that hypoxic MSCs enhanced axonal survival compared to normoxia culture conditions in a rat model of spinal cord injury [62].

The role of MSC priming with regard to pro-inflammatory cytokine has also been explored in the tissue regeneration. Indeed, as inflammation normally occurs after bone injury, it has been suggested that cytokine treatment might stimulate stem cell osteogenic differentiation. For instance, tumor necrosis factor alpha (TNF-α)-preconditioned CM from MSCs has been shown to improve bone regeneration in vitro by up-regulating bone morphogenetic protein 2 (BMP2) and thus stimulating cell proliferation and the expression of differentiation markers, such as runt-related transcription factor 2 (*Runx2*) and *Collagen I* [63]. Furthermore, the authors clearly demonstrated that the above described effects were associated with EXOs contained in the CM. In particular, TNF-α preconditioning stimulated MSCs to increase Wnt3a levels in the EXOs that, in turn, further improved cell proliferation and bone differentiation when compared to CM from unconditioned MSCs [64]. Regenerative properties of MSCs on bone were also shown by Novais et al. who demonstrated that both basic fibroblast growth factor (bFGF) and hypoxic priming improved MSCs proliferation and osteogenic differentiation resulting in the repair of critical size calvarial bone defects created in nude mice [65].

The priming of MSCs with small molecules also represents a promising therapeutic strategy for the treatment of neurodegenerative diseases. Indeed, the preconditioning of MSCs with cyclic AMP (cAMP), bFGF, platelet-derived growth factor (PDGF) and Heregulin β1 stimulates MSCs to secrete different neurotrophic factors (NTF), including glial-derived neurotrophic factor (GDNF), brain-derived neurotrophic factor (BDNF), VEGF and HGF [66]. In vivo studies showed that MSCs secreting NTF have protective effects in several animal models of neurodegenerative diseases, such as Parkinson’s disease, multiple sclerosis and Huntington’s disease [67,68,69], where, after MSCs administration, marked improvements of diseases were shown. Moreover, Linares et al. showed that preconditioning of MSCs with lithium and valproic acid (VPA) exerted neuroprotective effects. Actually, in transgenic mice with Huntington’s disease, primed MSCs administered intranasally migrated into the brain, improving motor and behavior performance, decreasing neuronal death and reducing huntingtin aggregates in the striatum [70].

In the field of regenerative medicine, a critical discussion is focused on the the fact that endogenous MSCs undergo senescence, with consequent reduction of the immunomodulatory and wound-healing properties of those cells [71,72]. These aging-related declines can be attributed to the intrinsic aging of stem cells [73] as well as to aging-related modifications of both the extracellular matrix and stem cell niche [74,75]. Overall, these tissue alterations reduce MSC self-renewal, maintenance and regenerative potential, and these dysfunctional processes have been implicated in frailty syndrome [76], which is characterized by declines in both health and function of organs/tissues. To date, there is no specific therapy available to prevent or treat frailty syndrome and, in this respect, MSCs could represent a potential tool to ameliorate or improve frailty. MSC treatments are safe in older/frail patients [77,78], where pro-inflammatory cytokines such as TNF-α, IL-6 and C-reactive protein (CRP) increased during aging [76]. Interestingly, it has been shown that, in an inflammatory environment typical of frailty patients, MSCs were primed to show anti-aging effects because they are able to reduce the expression of pro-inflammatory cytokines [79,80]. Moreover, some clinical trials (e.g., NCT02065245 and NCT04174898) investigated the safety and efficacy of intravenous infusion of MSCs as an innovative therapy for treating frailty patients and, in this setting, also testing the infusion of preconditioned MSCs to obtain superior results could be greatly interesting.

On the whole, these studies clearly suggest the importance of preconditioning MSCs in improving their impact on organ/tissue regeneration and repair. However, up to now little information has been available on the effects of preconditioned MSCs on parenchymal and tissue stem cells. We are still far from a clear understanding of the underlying molecular mechanisms, so it is now crucial to better define the paracrine factors released by MSCs that mediate the effects on proliferation and differentiation of tissue resident cells.

## 3. Main MSC Priming Strategies to Enhance the Production of Angiogenic Factors

MSCs became very appealing because of the clinical promise for tissue repair in regenerative medicine [52] and, for that reason, they are used in a high number of clinical trials [28]. The therapeutic benefits of MSCs have also been referred to the large number of molecules they secrete in response to specific stimuli, which then exert paracrine effects upon neighboring cells and tissues [81]. Among the therapeutic properties of MSCs, the angiogenic ones have been extensively studied because of their significance in many pathological conditions such as myocardial infarction, brain injury and limb ischemia [31,56,57]. The totality of soluble factors, mostly growth factors and cytokines, and including a vesicular component (including EXOs) carrying proteins, lipids and genetic material [82], is known as the secretome [83] and is considered to be responsible for the paracrine effects of MSCs on tissue regeneration processes including angiogenesis.

It has been postulated that the angiogenic potential of MSCs differs as a consequence of their original tissue (Wharton’s Jelly (WJ), AT and BM) due to the composition and concentration of angiogenic factors [30]. However, there are conflicting studies arguing that different sources of MSCs show different angiogenic effect. Previously, it has been reported that the AT-MSCs produce a higher expression of the angiogenic factors such as *VEGF*, insulin-like growth factor 1 (*IGF-1*) and *IL-8*, as well as matrix metalloproteinase-3 (*MMP3*) and *MMP9*, thus showing a greater angiogenic potential compared to BM-MSCs [84]. In contrast, a study published in 2019 on a proteomic analysis among different MSC secretomes assessed that BM-MSCs’ CM and WJ-MSCs’ CM retained higher angiogenic profiles when compared with the AT-MSCs’ CM because of higher expression of AKT serine/threonine kinase 1 (*AKT1*) and *bFGF* [85]. The fact that the paracrine activity is not consistent across different samples may be explained by the different sources (neonatal versus adult) and by a lack of standardization of culturing techniques. To solve these problems, in recent years, priming approaches to activate and generate MSC products with improved potential for different clinical applications have been investigated.

Among the soluble factors with angiogenic potential, MSCs secrete high levels of VEGF, transforming growth factor beta (TGF-β), HGF, IL-8, bFGF, monocyte chemoattractant protein 1 (MCP-1) and IL-6, as well as a lot of microvesicles carrying non-coding RNAs (ncRNAs) such as microRNAs (miRNAs) with angiogenic function [86,87]. Considering the in vivo MSC niche conditions that occur during tissue injury, hypoxia priming has been used as the main priming strategy to lead MSCs towards a pro-angiogenic phenotype [88,89,90,91,92,93,94,95,96].

Gorin et al. proved that bFGF and/or hypoxia can be considered as priming treatments capable of enhancing VEGF release and improving the angiogenic potential of MSCs [97]. Moreover, hypoxia-primed MSCs up-regulated VEGF and enhanced significantly angiogenesis when injected into the pulp cavities of rabbit molars [98]. Leroux et al. demonstrated that MSCs cultured under hypoxic conditions had increased high angiogenic and regenerative potentials via a paracrine wnt-dependent mechanism [58] and Xue et al. demonstrated that, in MSCs, hypoxia enhanced angiogenetic potential up-regulated the VEGF and protein kinase A (PKA) signaling pathway [99]. MSCs primed with hypoxia also showed increased expression of adhesion molecules, including fibronectin 1, E-cadherin, N-cadherin and integrins, crucial proteins for angiogenesis processes [100]. Moreover, hypoxic preconditioning increased MSC angiogenic properties via the HIF-1α-GRP78-Akt axis and an increased secretion of aforementioned factors such as VEGF, HGF and bFGF in a murine model of hindlimb ischemia [101]. Hypoxic priming increases hypoxia-inducible factor 1-alpha (HIF-1α) in MSCs that produce a conditioned medium enriched by VEGF that lead to migration and tube formation from HUVECs [102].

Interestingly, Han et al. showed that hypoxia treatment enhances MSC survival and their angiogenic properties through unusual mechanisms. In particular, the authors demonstrated that hypoxia preconditioning induced up-regulation of cellular prion protein (PrP^C^), which, in turn, enhanced MSCs proliferation via PrP^C^-dependent JAK2/STAT3 activation and inhibited oxidative stress-induced apoptosis via PrP^C^-dependent inactivation of cleaved caspase-3. Moreover, when those cells were administered to a murine hindlimb ischemia model, an improvement of functional recovery of the ischemic tissue and neovascularization were observed, and the levels of epidermal growth factor (EGF), VEGF, FGF and HGF were significantly higher in ischemic tissue treated with primed MSCs compared to control group [103].

As described above, secreted EXOs are another angiogenic action mechanism of MSCs [96,104] acting as cargo of miRNAs [105]. The importance of miRNAs as regulators for translation, RNA splicing and gene expression rely on the impact they can exert on host pathways [106]. Kane et al. in 2014 indicated miRNAs as being important regulators of angiogenesis [107] and many studies are related to the presence of miRNAs in MSCs’ secretome. Krawczenko et al. have recently demonstrated that four pro-angiogenic miRNAs, miR-126, miR-296, miR-378 and miR-210, were found to be highly expressed in microvescicles from MSCs, while there was a low expression of anti-angiogenic miRNAs such as miR-221, miR-222 and miR-92a [86]. Interestingly, in MSCs, hypoxia preconditioning induced up-regulation of miR-675 and subsequent angiogenic response by decreasing HIF-1α negative regulators and increasing VEGF secretion and VEGF receptor 1 expression [108]. Gonzalez-King and colleagues showed that MSCs overexpressing HIF-1α secrete higher amounts of EXOs compared to control MSCs, and these EXOs had increased angiogenic capacity and overexpressed different miRNAs implicated in angiogenesis processes, including miR-15, miR-16, miR-17, miR-31, miR-126, miR-145, miR-320a and miR-424 [109]. Furthermore, Zhu et al. demonstrated that hypoxia priming of MSCs induced the production of angiogenic EXOs that improved cardiac repair through miR-125 in a mice model of myocardial infarction [110]. There is very little information about MSC-derived ncRNAs modulating angiogenesis, and the strategy of priming MSCs to modulate functional ncRNAs to improve angiogenic potential of MSCs is very attractive.

## 4. Main MSC Priming Strategies to Enhance the Production of Immunomodulatory Factors

MSCs can modulate the functions of all immune cells, including T and B lymphocytes, natural killer cells, monocyte/macrophages, dendritic cells and neutrophils [111]. T and B cells, activated to execute their effector functions, are the primary mediators of different inflammatory and autoimmune diseases as well as being active in transplant rejection mechanisms and graft-versus-host disease [112]. In this context, MSCs exert a regulatory function on both T and B lymphocytes [113,114]. Concerning inflammatory cells, MSCs can reduce, or even inhibit neutrophils and macrophages infiltration [115], attenuate the cytotoxic activity of NK cells [116], inhibit the differentiation of dendritic cells [117] and induce the switch of monocytes from the pro-inflammatory M1 phenotype to an anti-inflammatory M2 phenotype [20].

There are several pieces of evidence that show that the immunomodulatory effects of MSCs may be mediated by paracrine factors, rather than a direct cellular action [86,92,93], and that the anti-inflammatory properties of MSCs can be enhanced to promote the efficacy of immunomodulation as a therapeutic strategy in the field of regenerative medicine.

The finest way of optimizing MSC action is their preconditioning (MSC priming), by which MSCs increase their survival rate and enhance their secretory activity [118]. The most commonly used way to prime MSCs for the improvement of immunomodulatory functions is their pre-activation with cytokines. MSCs primed with a variety of cytokines are enabled to produce different functional factors which exert specific immunomodulatory effects.

It has been shown that, using IL-17, MSCs increase the production of IL-6, which suppress T cell proliferation and inhibits Th1 cytokines production [119]. Instead, when primed with interferon gamma (IFN-γ), MSCs mainly produce indoleamide 2,3-dioxygenase (IDO) (a crucial factor able to suppress lymphocytes proliferation) [120], and also secrete key regulators of immunomodulation, such as prostaglandin E2 (PGE2), HGF, TGF-β and MCP-1. Furthermore, an increase of *MHC I* and *II* expression and of co-stimulatory molecules was also showed [121]. Interestingly, Sivanathan et al. proposed that unlike MSC-primed with IFN-γ, the priming with IL-17 enhances T cell immunosuppression but not their immunogenicity, showing no induction of MHC I/II and T cell co-stimulatory molecule CD40 [122]. In the same work, functional studies revealed that MSCs primed with IL-17 showed higher immunosuppressive potential against T cells proliferation not due to *IDO*, prostaglandin-endoperoxide synthase 2 (*COX-2*) or *TGF-β*, but due to increased *IL-6* expression [122]. Redondo-Castro et al. showed that when MSCs were primed simultaneously with IL-1α/β, TNF-α and IFN-γ, these cells were able to produce granulocyte colony-stimulating factor (G-CSF), a growth factor which has a strong anti-inflammatory effect on LPS-activated microglia cells [36]. English et al., simultaneously co-treating IFN-γ and TNF-α, induced MSCs to produce both IDO and PGE2, with the result of inhibiting in vitro the allogeneic mixed lymphocyte reaction (MLR) [123].

Preconditioned MSCs are also able to induce the polarization of monocytes towards an anti-inflammatory phenotype. Indeed, MSCs stimulated with the cocktail IFN-γ/TNF-α/LPS or with IFN-γ/IL-1β produced respectively PGE2 [124] or IL-6 [125], and both factors were able to induce the switch of monocyte from the pro-inflammatory M1 phenotype to an anti-inflammatory M2 phenotype. The same result has been obtained by priming MSCs only with IFN-γ. In this case, these cells produced EXOs containing different miRNAs (miR-23a, miR-26b, miR-125b, miR-130b, miR-140, miR-203a, miR-223, miR-224 and miR-320a) which not only act on monocyte polarization (switch of M1 phenotype to M2 phenotype), but also on T cells anergy induction [20]. Moreover, miRNAs delivered by EXOs that have both antiseptic and M2 monocytes polarization capacities have been shown to be produced by different kind of MSCs primed with IL-1β. Among these, miR-21 is produced by almost all IL-1β-primed MSCs [126] and miR-146a from UC-MSCs [127].

Apart from cytokines priming, specific culture conditions such as 3D cultures (cells grown as spheroids) can also affect MSC immunomodulatory activity. Indeed, MSCs grown as spheroids become able to produce high levels of IDO that has a strong in vitro antiproliferative effect on T cells [128]. Moreover, placenta-derived MSC spheroids secrete high levels of PGE2, HGF and leukemia inhibitory factor (LIF) [35] which can suppress pro-inflammatory M1 macrophages inducing M2 macrophages phenotypes [129,130,131]. Therefore, cytokine-mediated conditioning and/or 3D culture of MSCs may be considered as useful strategies to improve immunosuppressive properties maximizing the therapeutic effects of MSCs.

## 5. Main MSC Priming Strategies to Enhance the Production of Anti-Fibrotic Factors

Organ fibrosis represents the common consequence of functional cell replacement by fibrotic tissue, resulting in the reduction of the organ performance. Fibrosis involves many organs degenerating into numerous diseases, such as liver cirrhosis, kidney and myocardial fibrosis.

MSCs have been considered as a promising tool for treatment of various disorders including fibrosis. Indeed, while inflammation and fibrosis have a very close reciprocal relationship and MSCs have a strong immunomodulatory capacity, these cells appear to be tools capable of regulating fibrosis in many compartments.

The paracrine effect of MSCs in cardiac fibrosis was observed more than a decade ago, in a study in which MSC antifibrotic properties were linked to the regulation of matrix metalloproteinases (MMPs) [132]. In this work, Mias et al. showed that, in a rat model, MSC injection significantly improved morphological and functional cardiac parameters after myocardial infarction (MI). In particular, they demonstrated that CM from MSCs reduced the collagen secretion and increases the activity of MMP2 and MMP9 in cardiac fibroblasts. These processes prevent fibrosis by reducing collagen accumulation and consequently the fibrotic deposition of the extracellular matrix [132]. MSC effects have been also evaluated in a rat model of diabetic cardiomyopathy, in which uncontrolled diabetes was characterized by a long-term complication leading to myocardial fibrosis [133]. In this work, starting from the observation that PGE2 is secreted by MSCs during inflammation and immune response, the authors demonstrated the key role of this factor in fibrosis by using PGE2-deficient MSCs.

Interestingly, many MSCs priming methods, such as treatment with IFN-γ [20,121], IL-17 [122], TNF-α [134] and the growth of MSCs as spheroids [35,135], are able to up-regulate both MMPs and PGE2. Furthermore, different studies are evaluating hypoxia as a priming strategy to improve MSC potential in fibrosis inhibition [136]. In particular, a recent work in a mouse model of liver fibrosis demonstrated that hypoxia-primed MSCs (H-MSCs) enhanced *PGE2* expression [137]. They also showed increased levels of miR-210 in H-MSCs and this miRNA played different roles in fibrosis processes such as suppression of apoptosis, arrest of cell proliferation and repression of mitochondria respiration [137].

In an injured liver tissue, several pro-fibrotic factors such as TGF-β, PDGF and IL-4 are secreted by resident tissue cells or immune cells, playing a crucial role in the activation of hepatic stellate cells (HSCs), which are important for the production of extracellular matrix in the liver [138]. Moreover, macrophages also play a central role in liver fibrosis, in which during hepatic fibrogenesis, pro-inflammatory M1 macrophages secrete pro-fibrogenic factors such as TGF-β, PDGF and MCP-1 [139,140]. In the liver, antifibrotic activities of MSCs were attributed to either direct or indirect effects on HSCs. Indeed, MSCs can migrate towards liver injured sites where they are exposed to inflammatory cytokines and secrete many paracrine factors (e.g., PGE2, IDO, IL-6, IL-10), including EXOs, resulting in the suppression of immune cells that are responsible for the fibrosis process [141,142]. In this regard, it has been shown that MSCs primed with IFN-γ increased their production of PGE2, IDO, IL-6 and IL-10 and produced EXOs containing specific miRNAs capable of inducing immunomodulation by inhibiting PBMC proliferation and inducing the macrophage M2 phenotype [20]. Therefore, although it has not been proven yet, MSC priming with INF-γ could represent a very important strategy to enhance the therapeutic antifibrotic potential of MSCs in liver fibrosis.

Otherwise, genetic engineering has been used to potentiate the antifibrotic activity of MSCs. In particular, MSC-derived EXOs loaded with miR-19 have been used in MI mouse models [143]. This miRNA has been demonstrated to reduce cardiac fibrosis and enhance the recovery of cardiac function in mouse models [144]. In this way, priming methods such as hypoxic treatment have also been used to induce miR-125 expression in MSC-derived EXOs [110]. In particular, normoxia-conditioned MSC-derived EXOs (N-EXOs) and hypoxia-EXOs (H-EXOs) have been tested in MI mouse models. The MSC H-EXOs have an enhanced ability to recover the cardiac function when compared to N-EXOs and this gain of function was ascribed to the presence of EXO-derived miR-125. Indeed, after miRNA silencing (KO), the mice treated with mi-R125^ko^-H-EXOs showed an increase in the fibrotic area of the MI compared to normal H-EXOs EXOs [110]. The antifibrotic action of this miRNA has also been demonstrated in the liver where miR-125 reduces liver fibrosis by increasing the autophagy [145].

In vivo, liver injured tissue promotes inflammatory processes that stimulate MSCs to release various growth factors and cytokines such as HGF, EGF, IL-6 and TNF-α [146], and among them, HGF plays a well-established role in liver pathogenesis by attenuating liver fibrosis in various in vivo models [147,148,149,150]. Starting from this observation, engineered MSCs overexpressing HGF have been used in a rat model of liver fibrosis. In this model, the effect of modified MSCs was clearly enhanced compared to normal MSCs, with the consequent enhancement of antifibrotic activity [151]. A similar approach was used in an MI mouse model [152] and in a radiation-induced lung injury mouse model [153]. Another group also used the gene-modified MSCs to enhance the antifibrotic activity of these cells [154]. However, the genetic engineering approach to MSCs has some counter indications because these methods require genetic modification and are therefore incompatible with clinical applications. Also many papers showed with regard to this that different priming approaches, such as 3D cultures of MSCs [35], IFN-γ [121] and TNF-α [134] treatment, enhance the MSCs production of HGF, making them a potential therapeutic tool to treat liver fibrosis (Table 1). Very few studies explored the application of primed MSCs in the pathogenesis of fibrosis and this currently represents a scientific need in the field of MSC research.

## 6. Conclusions and Future Perspective

In the last decade, the growing scientific interest in MSCs has clearly shown that these cells may have a significant therapeutic potential that can be applied in the field of regenerative medicine. Their action appears to be mediated by the release of paracrine modulators that orchestrate tissue repair/regeneration in a wide variety of diseases and disorders. Indeed, the emerging idea is that the CM derived from MSCs or its components (for example EXOs) may itself be sufficient for therapeutic activity.

**Figure 2:**
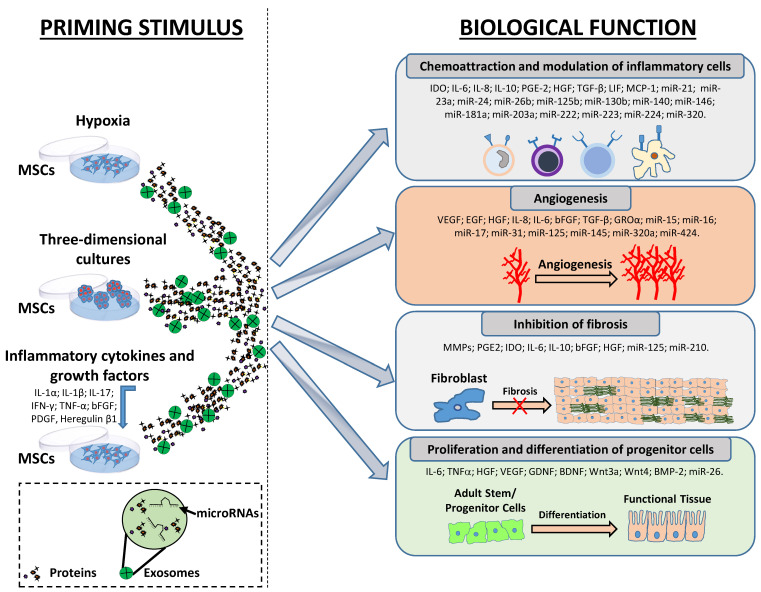
Schematic representation of the molecular effects after priming of MSCs. MSCs can be primed through different stimuli, including hypoxia, three-dimensional cultures, growth factors and cytokines, to enhance their therapeutic potential. In these cases, MSCs produce inducible regulatory factors such as growth factors, cytokines, chemokines and exosomes (which contain both proteins and microRNAs). Primed MSCs promote tissue regeneration/repair regulating different processes including inflammation and angiogenesis, the production of the extracellular matrix (ECM) and the regeneration of functional cells by differentiation of their progenitor cells.

There are currently a valuable number of clinical trials studying the effects of MSCs in many disorders (1213 studies at the time of writing, clinicaltrials.gov) and this number is increasing. However, clinical results suggest that MSCs have moderate or poor efficacy, thus being not very convincing in their applicability. MSC cultures might differ in their therapeutic capacity, and their heterogeneity also affects the MSC-derived secretomes that cause diverse effects on target cells. Therefore, it is essential that critical steps taken during the MSC production process should be more reproducible. Specific methods used to condition MSCs in stimulating their therapeutic functional properties could appropriately modify the therapeutic effects of the MSC secretome (Figure 2). For this reason, the strategy of strengthening the therapeutic potential of MSCs to direct their phenotype in therapeutically desirable ways is very appealing. Furthermore, the production of MSC-derived products provides a useful technology to enhance MSCs’ therapeutic potential and standardize the production of products intended for clinical use.

There is potential for improvement in MSC treatment and pretreating cells prior to use as therapeutic tool appear to be a promising strategy. A wide range of factors has been implicated in the paracrine therapeutic action of MSCs and further studies in this field must identify the best treatments and techniques that hold promise within specific disease models. Therefore, future experimental studies should define protocols for generation of MSC-derived products for each type of MSC population and for specific pathological conditions before MSC products can be applied in the field of regenerative medicine.

## Figures and Tables

**Table 1:** Priming treatments of MSCs and production of therapeutic factors.

MSCs	Priming Treatments	MSC Product	Functional Factors Detected	Biological Effects	Reference
AMSCs	IFN-γ	EXOs	miR-23a, miR-26b, miR-125b, miR-130b, miR-140, miR-203a, miR-223, miR-224, miR-320a	Regulation of T cell activation/anergy and induction of M2-like polarized phenotype in monocytes	[20]
AMSCs	3D cultures/spheroids	CM EXOs	HGF, PDGF, TGF-β, VEGF, FGF1, GROα, SDF-1, EGF, IL-6, PGE2, LIF	Increased angiogenesis and inhibition of PBMC proliferation	[35]
BM-MSCs	IL-1α/β; TNF-α; IFN-γ	CM	G-CSF	Reduction in the secretion of inflammatory mediators in LPS-activated microglial cells	[36]
AdMSCs	Hypoxia	CM	IL-6, TNF-α, HGF, VEGF	Induced liver regeneration	[56]
BM-MSCs	Hypoxia	CM	VEGF	Induced liver regeneration	[57]
BM-MSCs	Hypoxia	CM	Wnt4	Improvement of vascular and skeletal muscle fiber regeneration	[58]
BM-MSCs	Hypoxia	CM	-	Induced cardiomyocytes proliferation	[59]
MSCs	Hypoxia	EXOs	miR-26	Improvement of cardiomyocyte survival	[60]
BM-MSCs	Hypoxia	CM	HGF, VEGF	Improvement of neuronal cells proliferation	[61]
UC-MSCs	Hypoxia	CM	HGF, BDNF, VEGF	Improvement of axonal survival	[62]
AdMSCs	TNF-α	CM	BMP2	Improvement of bone regeneration	[63]
AdMSCs	TNF-α	EXOs	Wnt3a	Promoted the proliferation and osteogenic differentiation of primary osteoblastic cells	[64]
DP-MSCs	bFGF; Hypoxia	CM	-	Improvement of bone formation	[65]
BM-MSCs	cAMP, bFGF, PDGF, Heregulin β1	CM	GDNF, BDNF	Induction of striatal dopaminergic nerve terminals regeneration	[67]
BM-MSCs	cAMP, bFGF, PDGF, Heregulin β1	CM	GDNF, BDNF	Reduction of striatal volume changes associated with quinolinic acid lesions	[68]
BM-MSCs	Lithium, VPA	CM	-	Improvement of motor and behavior performance, and reduction of neuronal death and huntingtin aggregates in the striatum	[70]
DP-MSCs	bFGF; Hypoxia	CM	VEGF	Improvement of vascularization	[97]
DP-MSCs	Hypoxia	CM	VEGF	Improvement of vascularization	[98]
AdMSCs	Hypoxia	EXOs	-	Increase of migration and tube formation by HUVEC	[99]
AdMSCs	Hypoxia	CM	VEGF, HGF, bFGF	Increase of MSC angiogenic potential	[101]
WJ-MSCs	Hypoxia	CM	Angiogenin and VEGF	Increase of migration and tube formation angiogenesis	[102]
AdMSCs	Hypoxia	CM	EGF, VEGF, FGF, HGF	Improvement of functional recovery and neovascularization of the ischemic tissue	[103]
MSCs	Hypoxia	CM	VEGF	-	[108]
BM-MSCs	Hypoxia	EXOs	miR-125	Improvement of cardiac function	[110]
BM-MSCs	IL-17	CM	IL-6	Suppression of T cell proliferation; inhibition of both T cell activation and Th1 cytokines	[119]
AdMSCs BM-MSCs CB-MSCs	IFN-γ	CM	IDO	Suppression of human lymphocyte proliferation	[120]
BM-MSCs	IL-17	CM	IL-6	T cell immunosuppression	[122]
BM-MSCs	IFN-γ; TNF-α	CM	PGE2, IDO	Inhibition of allogeneic MLR	[123]
BM-MSCs	IFN-γ; LPS; TNF-α	CM	PGE2	Induction of monocytes polarization toward an anti-inflammatory M2 phenotype	[124]
BM-MSCs	IL-1β; IFN-γ	CM	IL-6	Induction of monocytes polarization toward an anti-inflammatory M2 phenotype	[125]
MSCs	IL-1β	EXOs	miR-21	Induced macrophage M2 polarization and ameliorates sepsis	[126]
UC-MSCs	IL-1β	EXOs	miR-146a	Amelioration of murine sepsis and induction of monocytes polarization toward an anti-inflammatory M2 phenotype	[127]
BM-MSCs	IFN-γ; Spheroids	CM	IDO	Suppression of T-cell activation and proliferation in vitro	[128]
BM-MSCs	Hypoxia	CM	PGE2, miR-210	Induced macrophage M2 polarization and ameliorates fibrosis	[137]

MSCs: mesenchymal stem cells; BM-MSCs: bone marrow-derived MSCs; AMSCs: amnion-derived MSCs; UC-MSCs: umbilical cord-derived MSCs; AdMSCs: adipose-derived MSCs; CB-MSCs: cord blood-derived MSCs; GMSCs: gingival-derived MSCs; WJ-MSCs: Wharton’s Jelly-derived MSCs; DP-MSCs: dental pulp-derived MSCs.

## Data Availability

Not applicable.

## Abbreviations

3D	Three-dimensional
AdMSCs	Adipose-derived MSCs
AKT1	AKT serine/threonine kinase 1
AMSCs	Amnion-derived MSCs
AT	Adipose tissue
BDNF	Brain-derived neurotrophic factor
bFGF	Basic fibroblast growth factor
BM	Bone marrow
BMP2	Bone morphogenetic protein 2
cAMP	Cyclic AMP
CB-MSCs	Cord blood-derived MSCs
CM	Conditioned medium
COX-2	Prostaglandin-endoperoxide synthase 2
CRP	C-reactive protein
DP-MSCs	Dental pulp-derived MSCs
EGF	Epidermal growth factor
EXOs	Exosomes
FGF1	Fibroblast growth factor 1
G-CSF	Granulocyte colony-stimulating factor
GDNF	Glial-derived neurotrophic factor
GMSCs	Gingival-derived MSCs
H-EXOs	Hypoxia-conditioned MSC-derived EXOs
HGF	Hepatocyte growth factor
HIF-1α	Hypoxia-inducible factor 1-alpha
H-MSCs	Hypoxia-primed MSCs
HSCs	Hepatic stellate cells
IDO	Indoleamide 2,3-dioxygenase
IFN-γ	Interferon gamma
IGF-1	Insulin-like growth factor 1
IL-6	Interleukin 6
LIF	Leukemia inhibitory factor
MCP-1	Monocyte chemoattractant protein 1
MHC I	Major histocompatibility complex
MI	Myocardial infarction
miRNAs	MicroRNAs
MLR	Mixed lymphocyte reaction
MMP3	Matrix metalloproteinase-3
MMPs	Matrix metalloproteinases
MSCs	Mesenchymal stromal/stem cells
ncRNAs	Non-coding RNAs
N-EXOs	Normoxia-conditioned MSC-derived EXOs
NTF	Neurotrophic factors
PCNA	Proliferating cell nuclear antigen
PDGF	Platelet-derived growth factor
PGE2	Prostaglandin E2
PKA	Protein kinase A
PrPC	Cellular prion protein
pSTAT3	Signal transducers and activators of transcription 3
Runx2	Runt-related transcription factor 2
SDF-1	Stromal cell-derived factor 1
SOCS3	Suppressor of cytokine signaling 3
TGF-β	Transforming growth factor beta
TNF-α	Tumor necrosis factor alpha
UC	Umbilical cord
VEGF	Vascular endothelial growth factor
VPA	Valproic acid
WJ	Wharton’s Jelly
